# Identification of *Aedes* mosquitoes by MALDI-TOF MS biotyping using protein signatures from larval and pupal exuviae

**DOI:** 10.1186/s13071-020-04029-x

**Published:** 2020-04-01

**Authors:** Amira Nebbak, Lionel Almeras

**Affiliations:** 1 Aix Marseille University, IRD, SSA, AP-HM, VITROME, Marseille, France; 2 Centre de Recherche Scientifique et Technique en Analyses Physico-Chimiques (CRAPC), Zone Industrielle, BP 384 Bou-Ismail, Tipaza, Algérie; 3 Unité Parasitologie et Entomologie, Département Microbiologie et Maladies Infectieuses, Institut de Recherche Biomédicale des Armées, 19–21 Boulevard Jean Moulin, 13005 Marseille, France; 4 IHU-Méditerranée Infection, Marseille, France

**Keywords:** *Aedes*, Identification, Exuviae, MALDI-TOF MS, Innovative strategy

## Abstract

**Background:**

Matrix-assisted laser desorption/ionization time-of-flight mass spectrometry (MALDI-TOF MS) biotyping is an innovative strategy, applied successfully for the identification of numerous arthropod families including mosquitoes. The effective mosquito identification using this emerging tool was demonstrated possible at different steps of their life-cycle, including eggs, immature and adult stages. Unfortunately, for species identification by MS, the euthanasia of the mosquito specimen is required.

**Methods:**

To avoid mosquito euthanasia, the present study assessed whether aedine mosquitoes could be identified by MALDI-TOF MS biotyping, using their respective exuviae. In this way, exuviae from the fourth-instar and pupal stages of *Aedes albopictus* and *Aedes aegypti* were submitted to MALDI-TOF MS analysis.

**Results:**

Reproducible and specific MS spectra according to aedine species and stage of exuviae were observed which were objectified by cluster analyses, composite correlation index (CCI) tool and principal components analysis (PCA). The query of our reference MS spectra database (DB) upgraded with MS spectra of exuviae from fourth-instar larvae and pupae of both *Aedes* species revealed that 100% of the samples were correctly classified at the species and stage levels. Among them, 93.8% (135/144) of the MS profiles reached the threshold log score value (LSV > 1.8) for reliable identification.

**Conclusions:**

The extension of reference MS spectra DB to exuviae from fourth-instar and pupal stages made now possible the identification of mosquitoes throughout their life-cycle at aquatic and aerial stages. The exuviae presenting the advantage to avoid specimen euthanasia, allowing to perform complementary analysis on alive mosquitoes.
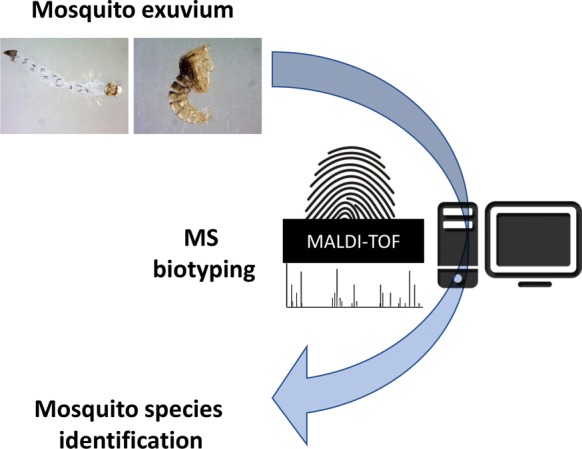

## Background

Mosquitoes (Diptera: Culicidae) are the common vectors of many infectious diseases distributed throughout the world [1]. Globalization has increased transport of goods and people between continents, and at the same time contributed to the global expansion of some invasive mosquito species [2]. Moreover, global warming facilitated the colonization of new areas by mosquitoes participating to the rapid change of the mosquito-borne diseases (MBDs) epidemiology [3, 4]. *Aedes* (*Ae.*) *albopictus* is a widespread species which is actually present in 25 European countries [5] and was considered as a major vector of arboviruses in Europe [6]. *Aedes aegypti* is also present in Europe and was responsible for a dengue outbreak in the region of Madeira which occurred in October 2012 [7]. Others mosquito species from the genus *Aedes* including *Ae. japonicus* (Japanese mosquito), *Ae. koreicus* (Korean mosquito) and *Ae. atropalpus* were reported as highly invasive species in Europe and are competent vectors for numerous human and animal pathogens [8]. These aedine species invaded several European countries like Switzerland [9], Germany [10], Austria [2], France [11] and Italy [12]. The establishment of these *Aedes* species in Europe participated to the emergence of autochthonous human cases of dengue and chikungunya [13, 14].

To face of this growing vulnerability of European countries to MBDs, the implementation of guidelines for surveillance and control of invasive mosquito was established [15]. However, a correct identification of mosquitoes to distinguish vectors from non-vectors is essential for the establishment of efficient control programmes and measuring the impact of vector control interventions [16]. In temperate areas, although integrated mosquito management is recommended, vector control concerns essentially larval stages [17]. For an efficient vector control at immature stages, accurate identification of mosquito species at the larval stage is compulsory. Morphological identification remains the primary method of mosquito classification. However, mosquito larval identification is generally complex and requires entomological expertise. Additionally, the lack of updated morphological criteria and dichotomic keys are factors hampering accurate identification of mosquitoes at larval stages [18]. The progress in molecular biology techniques during the last two decades has solved several limitations of morphological identifications. A recent study demonstrated that some mosquito sibling species could not be morphologically identified at immature stages due to character deviations which could be elucidated by genomic analyses [19]. Nevertheless, the molecular biology approach for mosquito identification remains laborious, time-consuming and costly [18].

Recently, MALDI-TOF MS biotyping emerged as an innovative and reliable tool for arthropod identification including mosquitoes [18, 20]. It was repeatedly demonstrated that MALDI-TOF MS biotyping could be useful for larvae and pupae identification also for specimens collected in the field [21, 22]. The current strategy for mosquito identification by this proteomic tool at the immature stages is done on whole specimens. The homogenization of entire larvae, prior to MALDI-TOF MS submission, does not allow complementary analyses such as punctual confirmation of specimen identity by molecular biology analyses. The organic buffer used for larvae grinding is incompatible with molecular biology assays [18]. Moreover, the euthanasia of whole larvae hinders assess to other mosquito parameters such as insecticide susceptibility or vector competence. Furthermore, the identification rate at the pupal stage is compromised by the phenomenon of insect metamorphosis which occurs at this last aquatic stage, altering the reproducibility and stability of the protein profiles [21]. The modification of the MS profiles hampers their reliable identification by perturbing spectral matching with reference MS spectra [22].

To circumvent these limitations, the aim of the present study was to assess whether MALDI-TOF MS biotyping could be applied to distinct mosquito species using exuviae from late-instar larvae or from pupae. In this way, the proof of concept was established using two laboratory-reared *Aedes* species, *Ae. albopictus* and *Ae. aegypti*.

## Methods

### Mosquitoes and exuviae collection

*Aedes aegypti* (Bora) and *Ae. albopictus* (Marseille strain, MRS) mosquitoes were laboratory-reared using standard methods [20]. The *Ae. albopictus* MRS strain originated from a field collection of larvae of Marseille city (south of France) [23]. The *Ae. aegypti* Bora strain was provided by the Dr. Fabrice Chandre, the scientific supervisor of the “Vectopole center” (IRD, Montpelier, France). Both species were reared in the same conditions as presented previously [23]. For egg production, blood-meals were given through a parafilm-membrane (Hemotek membrane feeding systems; Discovery Workshops, Accrington, UK) as previously described [21]. Larvae were reared until the pupal stage in trays containing 1 liter distilled water supplemented with fish food (TetraMinBaby; Tetra Gmbh, Herrenteich, Germany). Pupae were collected daily and transferred to a mosquito cage (Bug Dorm 1; Bioquip products, Compton, CA, USA). For the collection of exuviae, mosquito larvae at third-instar were transferred individually to a small jar and reared until the larvae molted into fourth-instar, pupae and adults. The exuviae from fourth-instar larvae and pupae were collected daily and rinsed with water prior to individual transfer in an Eppendorf tube. The remaining water was removed and the exuviae were immediately treated for MALDI-TOF MS analyses or frozen at − 20 °C until future MS submission. A total of 40 exuviae per species and stage were retrieved.

### Sample homogenization and MALDI-TOF MS analysis

Exuviae from fourth-instar and pupae were homogenized individually 3 × 1 min at 30 Hertz using a TissueLyser (Qiagen, Hilden, Germany) and glass beads of 1.0 mm diameter (product No. 11079110; BioSpec Products, Bartlesville, OK, USA) in 30 µl of homogenization buffer, composed of a mix (50/50) of 70% (v/v) formic acid (Sigma-Aldrich, Lyon, France) and 50% (v/v) acetonitrile (Fluka, Buchs, Switzerland), according to the standardized automated setting as described previously [24]. After sample homogenization, centrifugation was performed at 200×*g* for 1 min and 1 µl of the supernatant of each sample was spotted on the MALDI-TOF MS steel target plate in duplicate (Bruker Daltonics, Wissembourg, France). After air-drying, 1 µl of matrix solution composed of saturated α-cyano-4-hydroxycinnamic acid (Sigma-Aldrich), 50% (v/v) acetonitrile, 2.5% (v/v) trifluoroacetic acid (Sigma-Aldrich, Dorset, UK) and HPLC-grade water was added. To control matrix quality (i.e. absence of MS peaks due to matrix buffer impurities) and MALDI-TOF MS apparatus performance, matrix solution was loaded in duplicate onto each MALDI-TOF MS plate alone and with a bacterial test standard (reference No. 8255343; Bruker bacterial test standard).

### MALDI-TOF MS parameters

Protein mass profiles were obtained using a Microflex LT MALDI-TOF Mass Spectrometer (Bruker Daltonics, Hamburg, Germany), with detection in the linear positive-ion mode at a laser frequency of 50 Hz within a mass range of 2–20 kDa. The setting parameters of the MALDI-TOF MS apparatus were identical to those previously used [25].

### MS spectra analysis

MS spectra profiles were firstly controlled visually with flex Analysis v3.3 software (Bruker Daltonics). MS spectra were then exported to ClinProTools v2.2 and MALDI-Biotyper v3.0. (Bruker Daltonics) for data processing (smoothing, baseline subtraction, peak picking). MS spectra reproducibility was assessed by the comparison of the average spectral profiles (MSP, main spectrum profile) obtained from the two spots of each exuviae from fourth-instar larvae and pupae according to species with MALDI-Biotyper v3.0 software (Bruker Daltonics). MS spectra reproducibility and specificity were objectified using cluster analyses and composite correlation index (CCI) tool. Cluster analyses (MSP dendrogram) were performed based on a comparison of the MSP given by MALDI-Biotyper v3.0. software with clustering according to protein mass profile (i.e. their mass signals and intensities). The CCI tool from MALDI-Biotyper v3.0. software was also used, to assess the spectral variations within and between sample groups, as previously described [20, 26]. CCI matrix was calculated using MALDI-Biotyper v3.0. software with default settings (mass range 3.0–12.0 kDa; resolution 4; 8 intervals; auto-correction off). Higher correlation values (expressed by mean ± standard deviation, SD) reflecting higher reproducibility for the MS spectra, were used to estimate MS spectra distance between species for each exuviae stages (larval *vs* pupal). To visualize MS spectra distribution from *Aedes* species according to exuviae stage, principal components analysis (PCA) from ClinProTools v2.2 software was used with the default settings.

The top 5 of the most intense MS peaks per *Aedes* species using exuviae from immature stages were analyzed with ClinProTools software to estimate their performance to discriminate these species for each stage. The parameter settings in ClinProTools software for spectrum preparation were as follows: a resolution of 300; a noise threshold of 2.00; a maximum peak shift of 800 ppm and a match to calibrating agent peaks of 10%. Peak calculation and selection were performed on individual spectra with a signal-to-noise threshold of 2.00 and aggregation of 800 ppm. Based on the peak list obtained for each exuviae stage per species, the top 5 of the most intense m/z peaks were selected to include them into the genetic algorithm (GA) model. The selected peaks by the operator gave a recognition capability (RC) value together with the highest cross-validation (CV) value. The presence or absence of all discriminating peak masses generated by the GA model was controlled by comparing the average spectra from each species per body part.

### Database creation and blind tests

The reference MS spectra were created using spectra from fourth-instar and pupal exuviae of four specimens per species using MALDI-Biotyper software v3.0. (Bruker Daltonics) [27]. Reference MS spectra were created with an unbiased algorithm using the information on the peak position, intensity and frequency. MS spectra from the fourth-instar and pupal exuviae were tested against the in-house MS reference spectra database (DB), including mosquito legs and thoraxes reference MS spectra, but none from exuviae [20]. For all MS profiles queried against the MS DB, a matching is proposed. The reliability of species identification was estimated using the log score values (LSVs) obtained from the MALDI Biotyper software v.3.0, which ranged from 0 to 3. To distinct untrustworthy from reliable identification, a threshold value was established according to previous studies [27, 28]. LSVs greater than 1.8 were considered reliable for species identification. Data were analyzed by using GraphPad Prism software version 5.01 (GraphPad, San Diego, CA, USA). The raw data of exuviae MS spectra from *Ae. albopictus* and *Ae. aegypti* at fourth-instar and pupal stages which were included in the MS reference database are freely downloadable (Additional file 1).

## Results

### MS spectra of exuviae from the fourth-instar and pupal stages

**Fig. 1:**
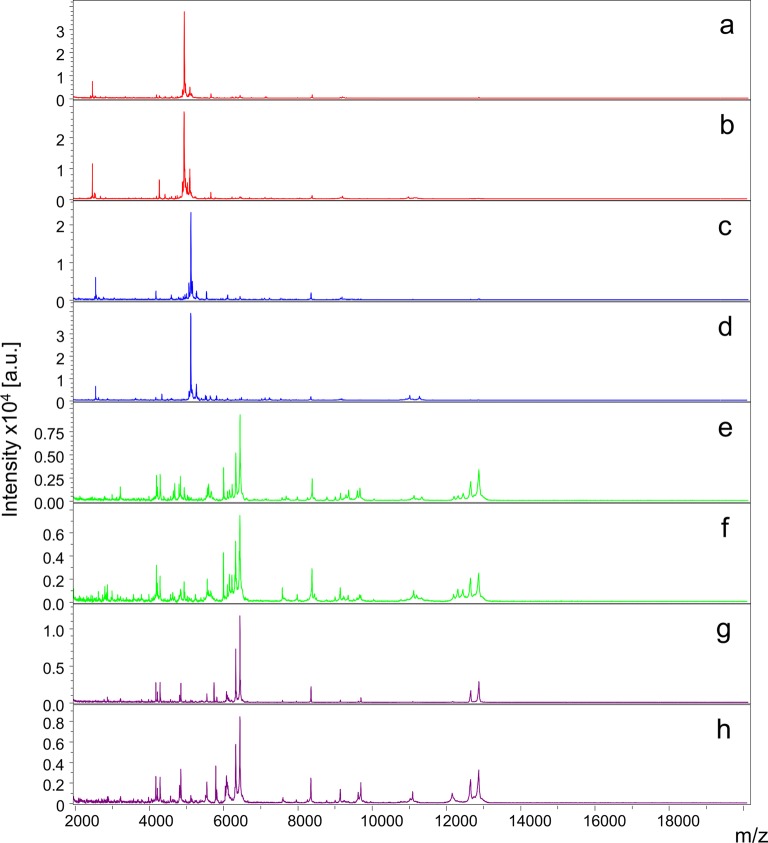
Comparison of MALDI-TOF MS spectra from exuviae of *Aedes* mosquitoes. Representative MS spectra of two exuviae from *Ae. albopictus* (**a**, **b**) and *Ae. aegypti* (**c**, **d**) at fourth-instar. MS spectra of two exuviae from *Ae. albopictus* (**e**, **f**), *Ae. aegypti* (**g**, **h**) at the pupal stage. *Abbreviations*: a.u., arbitrary units; m/z, mass-to-charge ratio

Forty exuviae from the fourth-instar and pupal stages per *Aedes* species (i.e*. Ae. albopictus* and *Ae. aegypti*) were submitted to MALDI-TOF MS analysis (Fig. 1). MS profiles of high intensity (> 2000 a.u.) were obtained for more than 95% (153/160) of exuviae from the two *Aedes* species. One MS peak was predominant in fourth-instar exuviae from *Ae. albopictus* and *Ae. aegypti* with mass to charge values of 4941.1 and 5118.6 daltons, respectively. However, the number of MS peaks detected between the exuviae stages was equivalent between the fourth-instar and pupal stages, with 85 ± 8 and 84 ± 6 MS peaks, respectively, irrespective of the species.

**Fig. 2:**
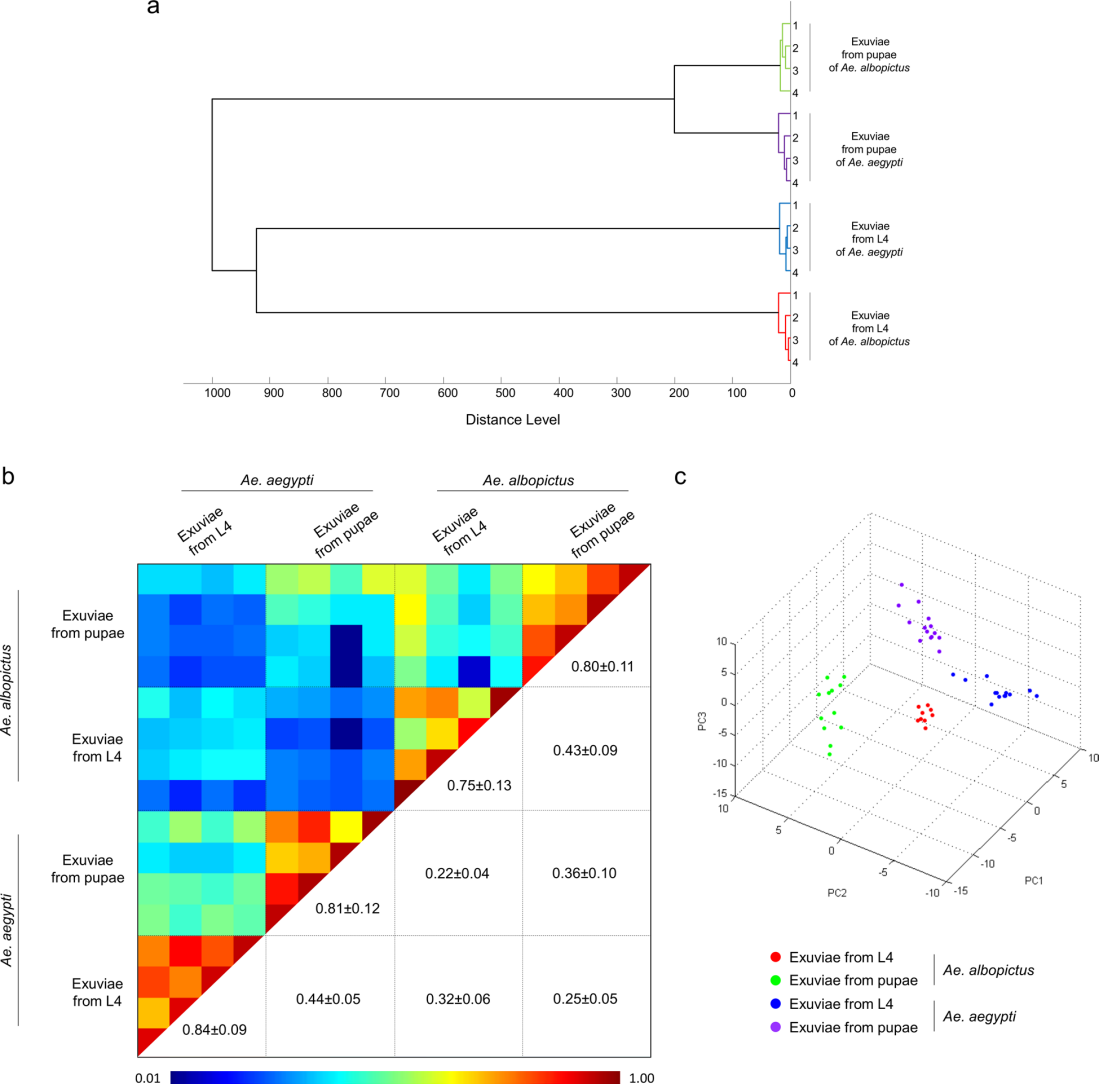
Reproducibility and specificity of MALDI-TOF MS spectra from exuviae of *Aedes* mosquitoes. **a** Four exuviae per species (i.e. *Ae. albopictus* and *Ae. aegypti*) and developmental stage (i.e. fourth-instar larvae and pupae) were used to construct the MSP dendrogram. The dendrogram was created using Biotyper v3.0 software and distance units correspond to the relative similarity of MS spectra. **b** The same four MS spectra per species and developmental stages were analysed using the composite correlation index (CCI) tool. Levels of MS spectra reproducibility are indicated in red and blue revealing relatedness and incongruence between spectra, respectively. The values correspond to the mean correlation coefficient and respective standard deviations obtained for paired condition comparisons. CCI were expressed as the mean ± standard deviation. **c** Principal components analysis (PCA) dimensional image from exuviae MS spectra of fourth-instar and pupae

To assess the reproducibility and specificity of MS spectra from fourth-instar and pupal exuviae according to species, cluster analysis was performed. Four samples per species and exuviae were used in the MSP dendrogram. Exuviae from the same species and developmental stage clustered on the same branch revealing the reproducibility and specificity of the protein profiles (Fig. 2a). The reproducibility of MS spectra was confirmed by CCI matrix highlighting the good correlation between exuviae of the same species and stage (mean ± SD: 0.80 ± 0.14; Fig. 2b). Additionally, lower CCI was obtained for the comparisons of MS spectra between *Aedes* species using exuviae from the fourth-instar and pupal stages (mean ± SD: 0.34 ± 0.10) sustained the species-specificity of protein profiles. The PCA revealed a clear separation of the dots corresponding to MS spectra from exuviae stages of *Aedes* species (Fig. 2c), comforting the specificity of MS profiles between these sample groups.

**Fig. 3:**
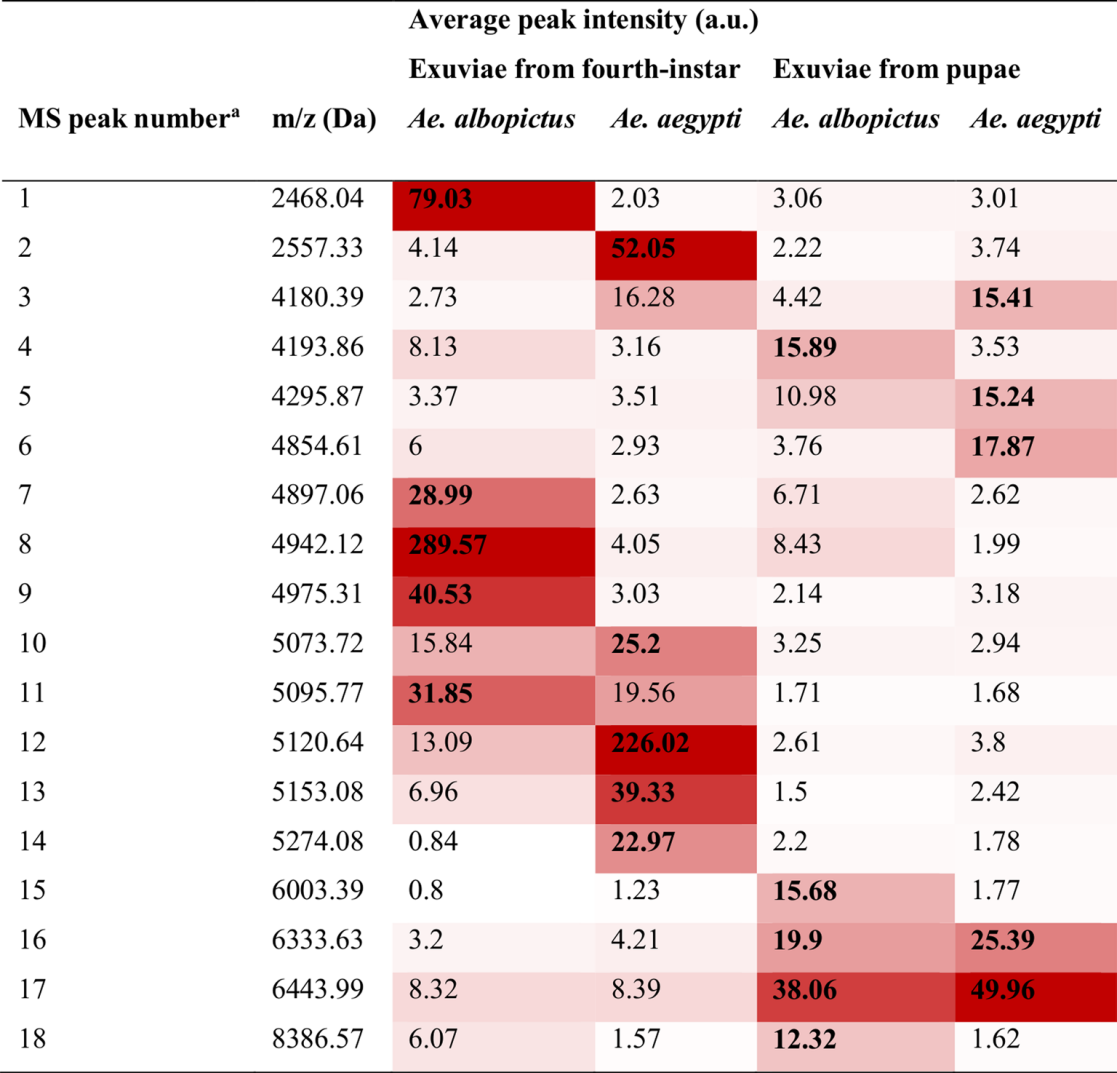
Top five mass peak list per *Aedes* species using exuviae from immature stages as biological material. The top five mass peaks per sample group are indicated in bold. Average MS peak intensity is indicated with using a colour scale, from white to red revealing moderate and elevate peak intensity, respectively. ^a^List of MS peaks used to distinct exuviae stages from two *Aedes* species based on the genetic algorithm model analysis of ClinProTools. *Abbreviations*: Da, daltons; m/z, mass to charge; a.u., arbitrary unit

As the intensity of MS spectra was a decisive criterion for correct sample classification by MS biotyping, we assessed whether the top five of the most intense mass peak lists of exuviae from fourth-instar larvae and pupae per species could be sufficient to distinguish these samples. As two MS peaks from exuviae of *Aedes* species were shared, a total of 18 MS peaks were selected corresponding to the top five mass peak lists per sample group (Fig. 3). To assess whether these 18 MS peaks could be discriminatory among these four groups, the MS peak list was included in the genetic algorithm (GA) model from ClinProTools 2.2 software. The combination of the presence/absence of these top five mass peak lists per stage of exuviae from *Aedes* species displayed, RC and CV values of 99.4% and 99.2%, respectively.

### MS reference spectra database creation and validation step

**Fig. 4:**
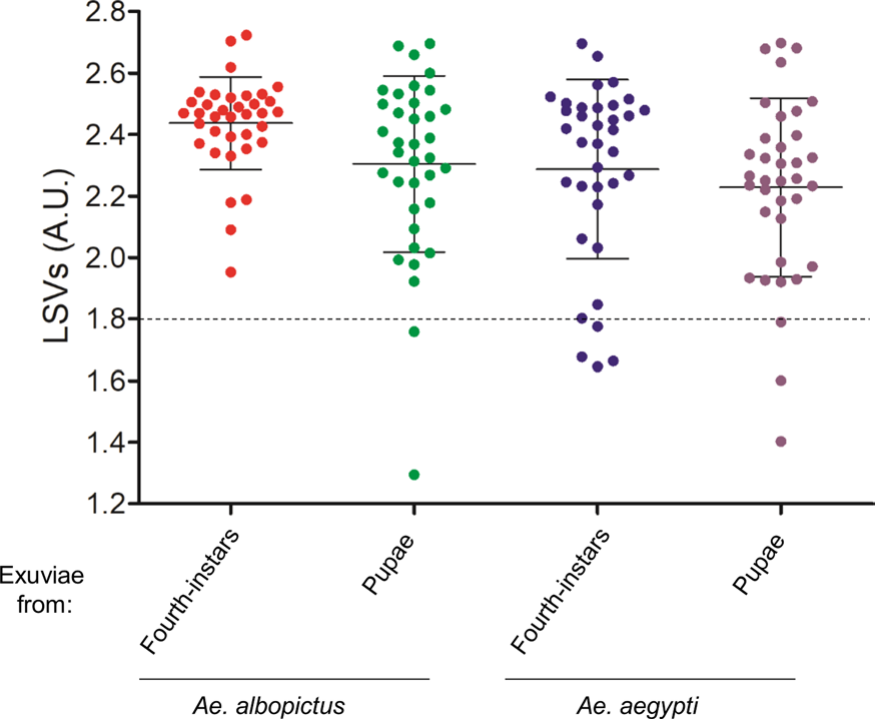
LSVs obtained following an in-house MS reference database query with MS spectra of *Ae. albopictus* and *Ae. aegypti* exuviae at fourth-instar and pupal stages. Horizontal dashed lines represent the threshold value for reliable identification (LSV > 1.8). *Abbreviations*: A.U., arbitrary units

The sixteen exuviae MS spectra, from fourth-instar larvae and pupae of both *Aedes* species used for MSP dendrogram analysis, were added to our MS spectra DB. This DB already included reference MS spectra of legs and thoraxes from 8 distinct mosquito species [20]. The 144 remaining MS spectra of exuviae from fourth-instar larvae and pupae of both *Aedes* species were queried against this upgraded reference MS DB. The LSVs ranged between 1.65–2.72 for fourth-instar exuviae and 1.23–2.70 for pupal exuviae (Fig. 4). 93.8% (135/144) of exuviae LSVs reached the threshold value (LSVs > 1.8) for reliable identification [24, 27], among which 100% of the samples were correctly classified at the species and stage levels. Among the nine MS spectra which did not reach the threshold LSVs, seven possessed MS profiles with lower intensity (< 2000 a.u.).

## Discussion

Unreliable species identification may contribute to unsuitable vector control efforts which could conduct to VBDs outbreaks. Reliable species identification is indispensable for adapting mosquito vector control strategies. MALDI-TOF MS biotyping emerged recently as a relevant alternative solution, to dispense with morphological and molecular biology identification tools [18].

This MS biotyping tool was successfully applied for the identification of mosquitoes at the adult [20, 29] but also at the immature stages [21, 22]. Nevertheless, the rates of correct identification at the pupal stages were lower than at late-instar stages using MALDI-TOF MS biotyping [22, 30]. At the beginning of the pupal cycle, MS profiles are shared with counter species MS spectra from late larval instar stages explaining specimen identification success [22]. The decrease of identification rate is attributed to the metamorphosis which occurs during pupal cycle [22]. Effectively, at the pupal stage, the transformation from an immature to an adult form conducts to protein repertoire evolution, hampering a species-specific MS spectra representative of this stage. Therefore, to counteract the constraints and limitations of the use of whole specimens at immature stages for MS submission, the analysis of mosquito exuviae from the aquatic stages appeared as an interesting compromise. The exuviae of the fourth-instar and pupal stages of *Ae. albopictus* and *Ae. aegypti* were then assessed as a template for species identification using MALDI-TOF MS biotyping.

The mosquito exuviae correspond to shedding of the exoskeleton and related structures. The analysis of exuviae MS spectra revealed that protein profiles are singular between these two aedine species but also between exuviae of the fourth-instar and pupal stages for each species. The reproducibility and specificity of MS spectra from fourth-instar and pupal exuviae according to species were highlighted by cluster analysis separating exuviae from the same species and developmental stage in the same branch and were objectified by CCI matrix. These results are similar to those previously obtained from mosquito larvae and pupae [22], as well as adult mosquitoes [24].

The singularity of the MS profiles between exuviae stages required the creation of reference MS spectra for the fourth-instar and pupal stages of each species. Approximately 300 structural cuticular proteins from *Anopheles gambiae* were recently found [31]. Among them, the expression of some cuticular proteins was restricted to a single metamorphic stage, which could explain the singularity of MS profiles between exuviae from the same species. In another study [32], the comparison of protein patterns of pupal cuticles and larval head capsules from *An. gambiae* on SDS-PAGE gel revealed distinct profiles with a major band of low molecular weight for larval samples. Likewise, the specificity of MS spectra according to the body part used from the same species was recently reported [20, 29]. The advantages of being able to query independently protein profiles from exuviae of fourth-instar larvae and pupae from the same specimens against the reference MS spectra DB, allow a double control for the identification of the mosquito. The consistency of the results are factors improving identification relevance and confidence. This cross-checking of specimen identification will be particularly imperative to distinguish closely related species with different vector competence living sympatrically [33].

Although 100% of the samples were correctly classified, MS spectra from nine samples (6.25%) did not reach the threshold (LSVs < 1.8) to consider identification reliable. The lower intensity (< 2000 a.u.) of these MS profiles conducted to imperfect matching with reference MS spectra. The sturdiness of the cuticle for to homogenization and the quick degradation of the exuviae are factors which could decrease the abundance of proteins and peptides for resulting MS spectra. The phenomena of protein degradation/digestion generating unstable MS profiles, was previously reported when blood meals from engorged mosquitoes were analyzed by MALDI-TOF MS approach [34].

Effectively, the lower protein abundance, for instance, in early larval stages [30] or eggs [35] from mosquitoes conducted to lower MS spectra, thus affecting the rate of reliable identification.

Mosquito exuviae were already used for morphological species identification [16, 36]. The use of exuviae for mosquito identification presents numerous advantages such as the preservation of the live specimen for future analyses, or the time saved, since no specimen dissection was required for its morphological classification. Although it is possible to find characters for separating mosquito species by meticulous examination of the pupal exuviae [37], this strategy remains laborious, reserved to specialists, and requiring undamaged samples. The absence of taxonomic keys and/or the overlapping features used for the exuviae, results in failures to reach definitive species identification based on morphology [16].

DNA-based identification methods could overcome the problems of intra-species feature variability or damaged field samples. Indeed, it has already been demonstrated that exuviae could be treated by molecular biology methods for specimen identification. Examples of such identification from exuviae include the differentiation of Neotropical anopheline species from the Amazon region [16]. Also, in Sri Lanka, the pupal exuviae have been used to differentiate molecularly sibling species of *An. subpictus* [19]. However, occasionally, exuviae collected directly from the field could not yield sufficient genomic DNA for amplification [38]. Failure in DNA amplification has been attributed to the decomposition of epithelial cells attached to the exuviae [38]. In the field, exuviae generally degrade quickly. Exuviae from fourth-instar are highly delicate and their collection in the field is very challenging. To detect aquatic organisms, the analysis of the environmental DNA (eDNA) from aquatic habitats remains one of the most efficient strategies [39]. This approach, elucidating water sample biodiversity by detecting persistent genetic material released by an organism into the environment, was recently applied to monitor invasive mosquito vectors [40]. However, the persistence of eDNA is directly linked to environmental factors [41, 42].

The present tool is not dedicated to list mosquito fauna from an aquatic site by analyzing their exuviae, but rather to identify specimens at immature stages collected in the field and reared to adulthood in the laboratory. The daily collection of larval and pupal exuviae allows to generate reproducible and specific MS spectra for each species, which are useful for specimen identification. The next step will be the validation of this innovative strategy on exuviae from aedine larvae collected in the field and laboratory bred until adulthood. The enlargement of the reference MS spectra DB from mosquito exuviae of other species will be also indispensable for the application of this MS tool as an alternative identification method. This strategy will overcome the limitations of morphological and molecular identification and will avoid the use of the entire body of the specimen, as it was done previously [22]. The situations in which this innovative approach will be interesting to use, are, for instance, each time it is necessary to quickly identify specimens at the aquatic stages without requiring to euthanize the mosquito. By keeping the specimen intact, complementary analysis on the same specimen is possible, such as insecticide susceptibility or vector competence. Henceforth, mosquito identification could then be done at a different development stage using MALDI-TOF MS biotyping, including pre-hatching [43], larval/pupal stages [22], imago [20] and now using their exuviae.

## Conclusions

Entomological surveillance of mosquito vectors becomes a necessity to deal with the rapid spread of MBDs, currently operating in different parts of the world. The present study demonstrated that MALDI-TOF MS biotyping could be extended to identification of fourth-instar larvae and pupal exuviae from aedine mosquitoes. This cost-effective, accurate and rapid approach is among the one of the best methods for specimen identification in periods consistent with the mosquito development cycle. The creation of reference MS spectra DB for mosquitoes covering the different developmental stages from eggs to the adult stage are essential for the wide use of this innovative proteomic tool. Moreover, the sharing and free access to reference MS spectra are also indispensable.

## Supplementary information

**Additional file 1.** MS spectra for raw exuviae from *Ae. albopictus* and *Ae. aegypti* at the fourth-instar and pupal stage added to the MS reference database. MS spectra were obtained using Microflex LT MALDI-TOF Mass Spectrometer (Bruker Daltonics).

## Data Availability

MS spectra from exuviae of aedine species at both stages used as reference MS spectra are included in Additional file 1.

## Abbreviations

CCI: Composite correlation index
CV: Cross-validation
GA: Genetic algorithm
LSVs: Log score values
MALDI-TOF MS: Matrix-assisted laser desorption/ionization time-of-flight mass spectrometry
PCA: Principal components analysis
RC: Recognition capability
